# The Quality of Registration of Clinical Trials: Still a Problem

**DOI:** 10.1371/journal.pone.0084727

**Published:** 2014-01-10

**Authors:** Roderik F. Viergever, Ghassan Karam, Andreas Reis, Davina Ghersi

**Affiliations:** 1 Department of Primary and Community Care, Radboud University Nijmegen Medical Center, Nijmegen, The Netherlands; 2 Department of Health Services Research and Policy, London School of Hygiene and Tropical Medicine, London, United Kingdom; 3 International Clinical Trials Registry Platform (ICTRP), Department of Ethics and Social Determinants of Health (ESD), World Health Organization, Geneva, Switzerland; 4 Department of Ethics and Social Determinants of Health (ESD), World Health Organization, Geneva, Switzerland; 5 NHMRC Clinical Trials Centre, Sydney Medical School, University of Sydney, Sydney, Australia; Johns Hopkins Bloomberg School of Public Health, United States of America

## Abstract

**Introduction:**

The benefits of clinical trials registration include improved transparency on clinical trials for healthcare workers and patients, increased accountability of trialists, the potential to address publication bias and selective reporting, and possibilities for research collaboration and prioritization. However, poor quality of information in registered records of trials has been found to undermine these benefits in the past. Trialists' increasing experience with trial registration and recent developments in registration systems may have positively affected data quality. This study was conducted to investigate whether the quality of registration has improved.

**Methods:**

We repeated a study from 2009, using the same methods and the same research team. A random sample of 400 records of clinical trials that were registered between 01/01/2012 and 01/01/2013 was taken from the International Clinical Trials Registry Platform (ICTRP) and assessed for the quality of information on 1) contact details, 2) interventions and 3) primary outcomes. Results were compared to the equivalent assessments from our previous study.

**Results:**

There was a small and not statistically significant increase from 81.0% to 85.5% in the percentage of records that provided a name of a contact person. There was a significant increase from 68.7% to 74.9% in the number of records that provided either an email address or a telephone number. There was a significant increase from 44.2% to 51.9% in the number of intervention arms that were complete in registering intervention specifics. There was a significant increase from 38.2% to 57.6% in the number of primary outcomes that were specific measures with a meaningful timeframe. Approximately half of all trials continued to be retrospectively registered.

**Discussion:**

There have been small but significant improvements in the quality of registration since 2009. Important problems with quality remain and continue to constitute an impediment to the meaningful utilization of registered trial information.

## Introduction

Clinical trials registration is now broadly considered an ethical and scientific responsibility.[1]–[8] In the past fifteen years, national and regional trial registries have been established in Africa, Asia, Australia/Oceania, Europe, North America and South America.[9] The WHO International Clinical Trials Registry Platform (ICTRP) was established in 2005 with the aim of bringing registered trial data from different trial registries together and creating a single point of access to information on all clinical trials conducted globally.[10] It now combines data from 15 national and regional clinical trial registries, offering access to data from more than 200,000 trials.

There are important advantages to the increased transparency on clinical trial conduct and reporting brought about by these developments. It improves access to information on clinical trials for healthcare workers, researchers and patients [11], [12]; it allows for steps to be taken against publication bias and selective reporting [2], [12]–[20]; it carries the potential to increase the accountability of those conducting clinical trial research; and it makes the identification of gaps in the health research landscape possible, thus facilitating priority setting in research [21]–[27].

The degree to which registered trial data can be used for these purposes depends on the completeness and meaningfulness of the data registered. The quality of data in registered records has been shown to be poor in the past.[2], [14], [15], [28]–[41] However, clinical trials registration has matured in recent years. Trialists may have gotten better at registering. Moreover, registries are likely to have improved their registration systems after the implementation of the International Standards for Clinical Trial Registries in 2010.[42]

This study was conducted to investigate whether the poor quality of registration observed in the past has been due to trial registration being in its nascence, or whether it is a more persistent problem. To do so, we repeated a study conducted by us in 2009, using the same methods and the same research team.[2]

## Methods

A random sample of 400 registered records of clinical trials that were registered between 1 January 2012 and 1 January 2013 was taken from the ICTRP database. Records of trials that were registered as having an observational study design were not eligible for the sample. For trials that were registered in more than one registry (duplicate records), only the record with the earliest registration date was considered eligible.[43] At the time the sample was taken the database included trials registered in fifteen different registries.[9]

### Sample size calculation

A sample size of 380 records was chosen, to ensure that all upper and lower 95% confidence intervals for extrapolation to the entire ICTRP dataset, calculated using the Wilson score interval (see further under analysis), would deviate 5% at most from the estimated number. A sample size of 380 also fulfilled this study's requirements to detect relatively minor changes in the quality of the three primary outcomes: the quality of contact details, interventions and outcomes (minor changes were defined as a 10% increase or decrease in the proportion of adequately registered records). It allowed for detecting an increase or decrease of 10% (using two-tailed test and α = 0.05) with β>0.85 in the quality of contact details and interventions and with β>0.95 in the quality of primary outcomes.

In our previous study in 2009, 3% of trials were incorrectly registered as interventional.[2] Therefore, a final sample size of 400 records was chosen to allow for exclusion of these trials.

### Data extraction

Registry name, trial ID, target sample size, inclusion criteria for gender and age of participants, recruitment status, date of registration, date of first enrolment and the public and scientific title for each record were downloaded from the ICTRP database and imported into Excel on 13 February 2013. Records were checked for the presence of entries in each of these fields.

All information that had to be extracted manually from the registered records was collected between 13 February 2013 and 23 February 2013. Information was always extracted manually from the complete registered record in the source registry.

Descriptive information on study design was extracted manually. Data on interventions and sponsorship was also extracted manually and was then coded. The system used to code interventions was adapted from the codes used for intervention types on ClinicalTrials.gov.[44] Primary sponsors were coded as being foundation, government, industry, university/hospital, or other. Trials were coded as being industry funded (primary sponsor was industry), partially industry funded (primary sponsor was non-industry, but secondary sponsor or source of monetary or material support was industry), or non-industry funded.

Records of trials that were registered as interventional but, during manual data extraction, turned out to be records of observational trials, diagnostic accuracy trials or treatment protocols for continuation of treatment after inclusion in a study protocol were excluded from further data extraction.

Descriptive statistics were generated for registry name, primary sponsor category, intervention type, study phase, study design, target sample size, randomization status and inclusion criteria for gender and age of participants. Registration dates and dates of first enrolment were compared to assess the degree of retrospective registration.

#### Contact information

The presence or absence of the following contact details was evaluated: name of a contact person (investigator or other), email address and telephone number. The WHO 20-item Trial Registration Data Set requires registration of separate scientific and public contact details.[45] There was, however, variation in registration formats for contact details between different registries. Some registries had one field for contact details, others had two separate fields for public and scientific contact details and others multiple contact fields. For records with only one contact field the presence of contact information was extracted from that field. For records with multiple contact fields, if the contact details were present in any of the fields, the information was denoted to be present.

#### Interventions

Given the considerable variability in the types of interventions evaluated in trials, comparison of registration quality between different intervention categories is difficult. Therefore, the evaluation of the quality of registered intervention data was limited to trials that investigated drugs, biologicals or vaccines, including active comparators. Placebo comparators were not evaluated. For each intervention and active comparator the presence or absence of the following five intervention specifics was collected: name, dose, duration of the intervention, frequency of administration and route of administration. All intervention arms were assessed separately. Name was denoted to be present if a company serial number or a drug name was provided. Only interventions and active comparators mentioned in the intervention field were assessed. Other texts in the record were scanned for additional information on mentioned interventions. To assess the overall completeness of registration of intervention specifics, a binary outcome variable was used that could be incomplete versus complete registration of the intervention. Complete registration entailed the reporting of drug name, dose, duration, frequency and route.

#### Outcome measures

The number of primary outcomes per record was collected. Each primary outcome was evaluated for specificity, using a classification system adapted from the system used by Zarin et al in their assessment of quality of outcomes.[30], [37] If a record contained multiple outcomes, all were assessed separately. Outcomes were classified as being a specific measure, a domain, vague, an unexplained abbreviation, or a part of safety monitoring.

Besides assessing the specificity of each outcome, the presence or absence of a time frame was collected for every outcome. Some outcomes assessed the duration of an event, the time to an event or were safety monitoring outcomes. For these outcomes, reporting a time frame is not possible, and the timeframe was therefore denoted as irrelevant. Time frames were denoted to be not meaningful when they did not specify a point in time when the outcome was to be measured.

Only outcomes mentioned in the outcome fields were assessed. Other texts in the record were scanned for additional information on mentioned outcomes.

To assess the overall quality of registration of primary outcomes, a binary outcome variable was used that could be registration of a specific measure with a meaningful time frame present or for which a time frame was irrelevant, versus any other outcome.

Finally, when there was more than one intervention (or active comparator) arm registered for a trial, or when there was more than one primary outcome registered, all intervention (and active comparator) arms and primary outcomes were assessed in this study. Multiple intervention arms and primary outcomes from one registered record are not independent. The effects of this non-independence on our reported outcomes are expected to be limited.

### Internal inconsistency in study design

Internal inconsistencies in study design fields were identified.[46] Internal inconsistencies were defined as records with multiple descriptors that were not compatible, such as “single-group” and “controlled or randomized”; “open-label” and “blinded”; and “double-blinded” without subject or investigator blinding.

### Assessment rules

The assessment rules and methods for data extraction for this study are analogous with the rules and methods used in our previous study on the quality of registration.[2] As then, all records were assessed for eligibility by RV who then extracted and coded the data. A more detailed overview of the rules used in all assessments is provided in the supporting file that accompanies our previous publication.[2]

### Analysis

95% confidence intervals (CI) were calculated for proportions of trials in the samples using continuity corrected Wilson score intervals with Singleton *et al.* adjustments for finite populations.[47]–[49] These 95% CIs reflect the confidence with which these proportions, measured in our samples of records, predict true proportions in the overall populations of all interventional trials on the ICTRP. The quality of registration was compared between trials registered between 17 June 2008 and 17 June 2009 [2] and trial registered between 1 January 2012 and 1 January 2013 using the Newcombe-Wilson test with continuity correction (with α = 0.05).[47]–[49]

This study intended to analyse changes in the quality of registration across all registries from 2008/2009 to 2012. However, the distribution of clinical trials across the registries changed from the former dataset to the latter, and several new registries were added to the ICTRP database. To be able to draw conclusions about changes in the quality of registration among the registries that were included in our first study, we conducted a sensitivity analysis for changes in data quality in the registered records from the three largest registries from 2008/2009 (ClinicalTrials.gov, ISRCTN and ANZCTR).

Statistical analyses were performed using MS Excel and SPSS 20.

## Results

**Figure 1:**
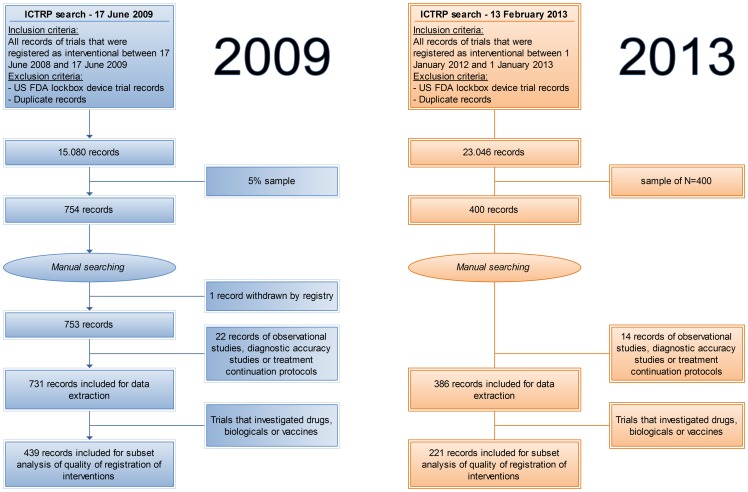
Flowcharts for the old 2009 study and for the new 2013 study.

A sample of 400 records was taken from a total of 23,046 unique interventional trials that were registered between 1 January 2012 and 1 January 2013. 14 records were excluded from data extraction because the corresponding trials were of an observational or diagnostic accuracy study design or were a treatment protocol for continuation of treatment after inclusion in a study protocol. A total of 386 records was included for data extraction, of which 221 (57.3% [52.2%–62.2%]) investigated drugs, biologicals or vaccines (Figure 1).

Baseline data on registry name, primary sponsor category, intervention type, study phase, study design, randomization status and inclusion criteria for gender of participants are presented in Table 1.

**Table 1:** General descriptive information from the two samples of clinical trials registered in 2008/2009 and in 2012.

	2008/2009		2012	
Category	Number of records	Percentage of records (%)	Number of records	Percentage of records (%)
**Registry name** 1				
ClinicalTrials.gov	628	85.9 [83.2–88.3]	222	57.5 [52.4–62.5]
JPRN	-	-	34	8.8 [6.3–12.2]
IRCT	4	0.5 [0.2–1.5]	31	8.0 [5.6–11.3]
ANZCTR	26	3.6 [2.4–5.2]	21	5.4 [3.5–8.3]
EU-CTR	-	-	21	5.4 [3.5–8.3]
ISRCTN	39	5.3 [3.9–7.3]	17	4.4 [2.7–7.1]
ChiCTR	11	1.5 [0.8–2.7]	14	3.6 [2.1–6.1]
CTRI	4	0.5 [0.2–1.5]	11	2.8 [1.5–5.2]
DRKS	2	0.3 [0.0–1.1]	5	1.3 [0.5–3.2]
NTR	16	2.2 [1.3–3.6]	4	1.0 [0.3–2.8]
CRiS	-	-	4	1.0 [0.3–2.8]
PACTR	-	-	1	0.3 [0.0–1.7]
RPCEC	-	-	1	0.3 [0.0–1.7]
SLCTR	1	0.1 [0.0–0.9]	0	0.0 [0.0–1.2]
ReBec	-	-	0	0.0 [0.0–1.2]
**Primary sponsor**				
Foundation	10	1.4 [0.7–2.6]	7	1.8 [0.8–3.9]
Government	39	5.3 [3.9–7.3]	15	3.9 [2.3–6.5]
Industry	246	33.7 [30.3–37.2]	97	25.1 [21.0–29.8]
University/hospital	398	54.4 [50.8–58.0]	245	63.5 [58.4–68.2]
Other^2^	37	5.1 [3.7–6.9]	19	4.9 [3.1–7.7]
Not specified	1	0.1 [0.0–0.9]	3	0.8 [0.2–2.4]
**Intervention type** 3				
Drug	385	52.7 [49.0–56.3]	197	51.0 [46.0–56.1]
Biological/vaccine	82	11.2 [9.1–13.7]	34	8.8 [6.3–12.2]
Device	49	6.7 [5.1–8.8]	31	8.0 [5.6–11.3]
Procedure/surgery	69	9.4 [7.5–11.8]	35	9.1 [6.5–12.5]
Radiation	23	3.1 [2.1–4.7]	6	1.6 [0.6–3.5]
Behavioural	76	10.4 [8.4–12.9]	39	10.1 [7.4–13.6]
Genetic^4^	14	1.9 [1.1–3.3]	4	1.0 [0.3–2.8]
Dietary supplements	53	7.3 [5.5–9.4]	21	5.4 [3.5–8.3]
Physical therapy	23	3.1 [2.1–4.7]	18	4.7 [2.9–7.4]
Organizational	21	2.9 [1.8–4.4]	15	3.9 [2.3–6.5]
Diagnostic	9	1.2 [0.6–2.4]	11	2.8 [1.5–5.2]
Other	16	2.2 [1.3–3.6]	11	2.8 [1.5–5.2]
**Study phase** 5				
0	10	1.4 [0.7–2.6]	4	1.0 [0.3–2.8]
I	106	14.5 [12.1–17.3]	48	12.4 [9.4–16.2]
I & II	38	5.2 [3.8–7.1]	21	5.4 [3.5–8.3]
II	122	16.7 [14.1–19.6]	57	14.8 [11.5–18.8]
II & III	16	2.2 [1.3–3.6]	9	2.3 [1.1–4.5]
III	101	13.8 [11.5–16.5]	43	11.1 [8.3–14.8]
IV	85	11.6 [9.5–14.2]	40	10.4 [7.6–13.9]
Not specified	253	34.6 [31.2–38.2]	164	42.5 [37.5–47.6]
**Study design**				
Single arm	162	22.2 [19.3–25.3]	85	22.0 [18.1–26.5]
Controlled	458	62.7 [59.1–66.1]	269	69.7 [64.8–74.2]
Crossover	79	10.8 [8.7–13.3]	31	8.0 [5.6–11.3]
Not specified	32	4.4 [3.1–6.2]	1	0.3 [0.0–1.7]
**Randomization**				
Randomized	518	70.9 [67.4–74.1]	283	73.3 [68.6–77.6]
Non-randomized	23	3.1 [2.1–4.7]	11	2.8 [1.5–5.2]
Not specified	29	4.0 [2.7–5.7]	7	1.8 [0.8–3.9]
Not applicable	161	22.0 [19.1–25.2]	85	22.0 [18.1–26.5]
**Gender**				
M	39	5.3 [3.9–7.3]	23	6.0 [3.9–8.9]
F	79	10.8 [8.7–13.3]	44	11.4 [8.5–15.1]
Both	599	81.9 [79.0–84.6]	308	79.8 [75.4–83.6]
Not specified	14	1.9 [1.1–3.3]	11	2.8 [1.5–5.2]
**Total per category**	**731**	**100%**	**386**	**100%**

^1^ The number of registries that provide data to the ICTRP has increased from nine to fifteen in between 2008/2009 and 2012. Registry acronyms stand for: ClinicalTrials.gov (CT.gov), Japan Primary Registries Network (JPRN), Iranian Registry of Clinical Trials (IRCT), Australian New Zealand Clinical Trials Registry (ANZCTR), EU Clinical Trials Register (EU-CTR), International Standard Randomized Controlled Trial Number Register (ISRCTN), Chinese Clinical Trial Register (ChiCTR), Clinical Trials Registry - India (CTRI), German Clinical Trials Register (DRKS), The Netherlands National Trial Register (NTR), Clinical Research Information Service (CRiS) Republic of Korea, Pan African Clinical Trial Registry (PACTR), Cuban Public Registry of Clinical Trials (RPCEC), Sri Lanka Clinical Trials Registry (SLCTR) and Brazilian Clinical Trials Registry (ReBec).

^2^ Other sponsors consisted of persons that were registered as primary sponsor, non-governmental organizations, collaborative research institutions and clinical research organizations.

^3^ Overlap was possible, total in this category was greater than 731 in 2008/2009 and greater than 386 in 2012.

^4^ Genetic interventions consisted of gene transfer therapy and somatic cell transplants.

^5^ The presence of study phase in records was analysed separately for trials in drugs, biologicals or vaccines. 2008/2009: Of 439 trials researching these types of interventions, study phase was reported in 370 records (84.3%). 2012: Of 221 trials researching these types of interventions, study phase was reported in 172 records (77.8%).

Records were additionally checked for the presence of entries in the fields for recruitment status, date of first enrolment and the public and scientific title. The former three were present in all records, the latter was reported in 379 records (98.2% [96.1%–99.2%]), which constituted a significant improvement from the observed 95.8% in 2008/2009. Furthermore, information was collected on sample size and age of participants. Sample size was reported in 384 records (99.5% [97.9%–99.9%]), which was not statistically different from the observed 98.6% in 2008/2009. The median target sample size was 77 [IQR 39–200]. Age of participants was reported in 375 records (97.2% [94.8%–98.5%]), which was not statistically different from the observed 95.8% in 2008/2009. 56 records (14.5% [11.2%–18.5%]) mentioned inclusion of participants <18 years of age. Finally, registration dates and dates of first enrolment were compared. The majority of records in our sample did not provide a day for the date of first enrolment but only a month and a year, which limited this analysis to comparing the month in which trials were registered to the month in which the first participant was recruited. The registration date was in a later month than the date of first enrolment in 185 records (47.9% [42.9%–53.0%]), which was not statistically different from the observed 53.4% in 2008/2009. This difference was more than one month in 158 records (40.9% [36.0%–46.0%]), which was not statistically different from the observed 43.6% in 2008/2009. The median of the difference was 8 months. Registration date and date of first enrolment were in the same month in 76 records (19.7% [15.9%–24.1%]). The registration date was in an earlier month than the date of first enrolment in 125 records (32.4% [27.8%–37.3%]). The median of this difference was 2 months.

### Quality of registration of contact information

Overall, 330 records reported a name of a contact person (85.5% [81.5%–88.8%]). 259 records provided an email address (67.1% [62.2%–71.7%]) and 272 records a telephone number (70.5% [65.6%–74.9%]). 289 records provided either an email address or a telephone number (74.9% [70.2%–79.0%]). These constituted significant improvements as compared to 2008/2009 for the presence of an email address, the presence of a telephone number and the presence of either (Table 2). Improvement in the presence of a name of a contact person was not significant. All changes for the subcategories of industry, non-industry and partially industry sponsored records were not significant. Contact details remained present less frequently among industry sponsored trials than among non-industry sponsored trials.

**Table 2:** The presence of contact details in registered records in 2008/2009 and 2012.

Sponsorship	Year	N	Name (%)	Email (%)	Telephone nr. (%)	Email or tel. nr. (%)
*Industry*	*2008/9*	246	53.7 [47.3–59.9]	39.0 [33.0–45.4]	46.7 [40.5–53.1]	56.5 [50.1–62.7]
	*2012*	97	53.6 [43.3–63.6]	47.4 [37.3–57.7]	57.7 [47.3–67.5]	61.9 [51.4–71.3]
*Partially industry*	*2008/9*	76	97.4 [90.1–99.5]	63.2 [51.4–73.6]	65.8 [54.1–75.9]	65.8 [54.1–75.9]
	*2012*	25	96.0 [77.8–99.8]	72.0 [50.5–87.1]	84.0 [63.2–94.7]	84.0 [63.2–94.7]
*Non-industry*	*2008/9*	408	94.4 [91.6–96.3]	70.8 [66.2–75.1]	74.3 [69.7–78.3]	76.5 [72.1–80.4]
	*2012*	261	96.2 [92.9–98.0]	73.6 [67.7–78.7]	73.6 [67.7–78.7]	78.5 [73.0–83.2]
*Overall*	*2008/9*	731^1^	81.0 [78.0–83.7]	59.4 [55.7–62.9]*	64.2 [60.6–67.6]*	68.7 [65.2–72.0]*
	*2012*	386^1^	85.5 [81.5–88.8]	67.1 [62.2–71.7]*	70.5 [65.6–74.9]*	74.9 [70.2–79.0]*

Legend Table 2: Percentages of records for which different aspects of contact details were present in 2008/2009 and 2012.

*  =  significant difference between 2008/2009 and 2012.

^1^ Numbers of records for subcategories do not add up to total because in 2008/2009 for one trial no primary sponsor was registered and in 2012 for three trials no primary sponsor was registered.

The presence of contact details was disaggregated according to trials' recruitment status (Table 3). The presence of names of contact persons did not differ markedly for trials with a different recruitment status, but email addresses, telephone numbers or either were present more frequently among recruiting or not yet recruiting trials than among completed trials, especially for industry sponsored trials.

**Table 3:** The presence of contact details according to recruitment status for trials registered in 2012.

Sponsorship	Recruitment status	N	Name (%)	Email (%)	Telephone nr. (%)	Email or tel. nr. (%)
*Industry*	*Not yet recruiting*	10	80.0 [44.4–96.4]	80.0 [44.4–96.4]	70.0 [35.5–91.8]	80.0 [44.4–96.4]
	*Recruiting*	51	47.1 [33.2–61.3]	66.7 [52.0–78.8]	84.3 [70.9–92.5]	90.2 [77.9–96.3]
	*Completed or stopped*	36	55.6 [38.4–71.6]	11.1 [3.6–26.9]	16.7 [7.0–33.4]	16.7 [7.0–33.4]
*Partially industry*	*Not yet recruiting*	9	100.0 [63.1–100.0]	100.0 [63.1–100.0]	100.0 [63.1–100.0]	100.0 [63.1–100.0]
	*Recruiting*	13	100.0 [71.8–100.0]	61.5 [32.4–84.8]	84.6 [53.8–97.3]	84.6 [53.8–97.3]
	*Completed or stopped*	3	66.7 [12.7–98.2]	33.3 [1.8–87.3]	33.3 [1.8–87.3]	33.3 [1.8–87.3]
*Non-industry*	*Not yet recruiting*	51	100.0 [91.3–100.0]	88.2 [75.5–95.1]	94.1 [82.8–98.5]	96.1 [85.5–99.3]
	*Recruiting*	144	95.1 [89.9–97.8]	79.2 [71.5–85.3]	79.2 [71.5–85.3]	85.4 [78.4–90.5]
	*Completed or stopped*	66	95.5 [86.5–98.8]	50.0 [37.6–62.4]	45.5 [33.4–58.1]	50.0 [37.6–62.4]
*Overall*	*Not yet recruiting*	70^1^	97.1 [89.2–99.5]	88.6 [78.2–94.6]	91.4 [81.7–96.5]	94.3 [85.3–98.1]
	*Recruiting*	208^1^	83.7 [77.8–88.2]	75.0 [68.5–80.6]	80.8 [74.6–85.7]	86.5 [81.0–90.7]
	*Completed or stopped*	105^1^	81.0 [71.9–87.7]	36.2 [27.2–46.2]	35.2 [26.4–45.2]	38.1 [29.0–48.1]

Legend Table 3: Percentages of records for which different aspects of contact details were present for recruiting and not-recruiting trials.

^1^ Numbers of records for subcategories do not add up to 386 because for three trials no primary sponsor was registered.

Sensitivity analysis among the three largest registries showed effects that were congruent with the changes found in the full dataset. From 2008/2009 (693 trials) to 2012 (260 trials), reporting improved from 79.9% to 86.2% for the name of a contact persons, from 57.9% to 61.9% for an email address, from 62.5% to 67.7% for a telephone number, and from 67.2% to 70.8% for the presence of either.

### Quality of registration of interventions involving drugs, biological or vaccines

There were 221 records of trials that investigated drugs, biologicals or vaccines. These reported 351 experimental or active comparator arms (Table 4). Completeness of registration of the name of the intervention, the duration of the intervention, the frequency of administration and the route of administration did not significantly change between 2008/2009 and 2012. Information on the dose was present significantly more often in 2012 than in 2008/2009. 182 arms (51.9% [46.5%–57.1%]) were complete in registering intervention specifics, which also constituted a significant improvement from the observed 44.2% in 2008/2009.

**Table 4:** The completeness of intervention specifics in registered records in 2008/2009 and 2012.

Year	N	Name (%)	Dose (%)	Duration (%)	Frequency (%)	Route (%)	All complete (%)
*2008/9*	726	98.2 [96.9–99.0]	70.5 [67.1–73.8]*	70.0 [66.5–73.2]	75.8 [72.5–78.8]	73.7 [70.3–76.8]	44.2 [40.6–47.9]*
*2012*	351	96.6 [94.0–98.1]	77.5 [72.7–81.7]*	68.9 [63.8–73.7]	73.2 [68.2–77.7]	79.2 [74.5–83.2]	51.9 [46.5–57.1]*

Legend Table 4: Percentages of total number of intervention (and active comparator) arms for which different intervention specifics were present in 2008/2009 and 2012.

*  =  significant difference between 2008/2009 and 2012.

Sensitivity analysis showed small improvements for the completeness of registration of all intervention characteristics in registered records from the three largest registries. From 2008/2009 (696 intervention arms) to 2012 (217 intervention arms), reporting improved from 98.9% to 100.0% for the name of the drug, from 71.3% to 82.0% for dose, from 71.0% to 79.3% for duration, from 76.7% to 84.3% for frequency, and from 74.7% to 84.8% for route. The proportion of arms that were complete in registering interventions specifics rose from 44.7% to 57.6%.

### Quality of registration of outcome measures

The 386 included trial records reported 705 primary outcomes. 261 records (67.6% [62.7%–72.2%]) reported one primary outcome, 62 (16.1% [12.6%–20.2%]) reported two, 29 (7.5% [5.2%–10.7%]) reported three and 32 (8.3% [5.8%–11.6%]) reported four or more. The maximum number of primary outcomes reported in one record was 52. Two records (0.5% [0.1%–2.1%]) reported no primary outcome at all.

The degree of specificity of reported outcomes was assessed (Table 5). To prevent skewing of the data, the outcomes in the record with 52 outcomes were counted as one for this analysis (the 2^nd^ highest number of outcomes in any record was 12). 377 primary outcomes (57.6% [53.8%–61.4%]) were specific measures for which a meaningful time frame was present or for which a time frame was irrelevant. This constituted a significant improvement from the observed 38.2% in 2008/2009.

**Table 5:** Degree of specificity of primary outcomes in 2008/2009 and 2012.

Classification	2008/9 (N = 1271)	2012 (N = 654)	Examples
Specific measure (%)	47.1 [44.4–49.9]*	69.1 [65.4–72.6]*	All-cause mortality, quality of life by SF-36, pulmonary functioning by FEV-1
Domain (%)	36.7 [34.1–39.4]*	21.1 [18.1–24.5]*	Freedom from progression, quality of life, pulmonary functioning
Vague (%)	5.4 [4.3–6.8]*	3.2 [2.1–4.9]*	Efficacy, symptoms, laboratory parameters
Unexplained abbreviation (%)	3.5 [2.6–4.6]*	1.2 [0.6–2.5]*	Any unexplained abbreviation
Safety monitoring (%)	7.3 [6.0–8.9]	5.4 [3.8–7.4]	Adverse event monitoring, drug toxicities, complications
**Time**			
Time present (%)	65.9 [63.3–68.5]	63.3 [59.5–67.0]	Mortality at one year
Time present, not meaningful (%)	10.8 [9.2–12.6]*	7.6 [5.8–10.0]*	ECG twice a year, social impact throughout study
Time absent (%)	7.7 [6.3–9.3]*	13.8 [11.3–16.7]*	
Time irrelevant (%)	15.6 [13.7–17.7]	15.3 [12.7–18.3]	Duration of stay in ICU, time to progression

Legend Table 5: The specificity and presence of a time frame for primary outcomes, presented as percentages of the total number of primary outcomes in 2008/2009 and 2012.

*  =  significant difference between 2008/2009 and 2012.

Sensitivity analysis also showed improvements for the quality of reported primary outcomes in registered records from the three largest registries. From 2008/2009 (1186 primary outcomes) to 2012 (401 primary outcomes), the proportion of primary outcomes that were specific measures for which a meaningful time frame was present or for which a time frame was irrelevant improved from 38.5% to 66.1%.

### Internal inconsistencies in study design

Internal inconsistencies in the study design fields were encountered in 10 records (2.6% [1.3%–4.9%]). This was a significant improvement from the observed 9.3% in 2008/2009.[46]

### Differences between registries

Differences between registries in the quality of information on contact details, interventions and primary outcomes were assessed (Table 6). Only registries with more than ten records, intervention arms or outcomes, respectively, were included for this comparison. There are differences between registries in the quality of reporting, yet there are few that score good on all aspects of quality, or bad on all aspects.

**Table 6:** The quality of information on contact details, interventions and primary outcomes per registry for trials registered in 2012.

	Contact details		Intervention	Primary outcomes
	*Name present (%)*	*Email or tel.nr. present (%)*	*All intervention specifics complete (%)*	*Outcome was a specific measure with a meaningful or irrelevant timeframe (%)*
**Registry name** 1				
ClinicalTrials.gov	83.8 [78.1–88.2]	68.9 [62.4–74.8]	54.0 [46.9–61.1]	68.6 [63.4–73.4]
JPRN	91.2 [75.3–97.7]	58.8 [40.9–74.8]	17.6 [7.4–35.1]	13.8 [6.6–25.9]
IRCT	100.0 [86.4–100.0]	100.0 [86.4–100.0]	65.6 [46.9–80.8]	76.7 [65.2–85.4]
ANZCTR	100.0 [80.9–100.0]	100.0 [80.9–100.0]	100.0 [73.4–100.0]	76.9 [56.0–90.2]
EU-CTR	19.0 [6.3–42.5]	85.7 [62.8–96.2]	14.3 [4.7–33.5]	69.0 [49.1–84.0]
ISRCTN	100.0 [77.2–100.0]	58.8 [33.6–80.5]	– 1	25.0 [11.5–45.1]
ChiCTR	100.0 [73.4–100.0]	100.0 [73.4–100.0]	0.0 [0.0–34.2]	0.0 [0.0–14.0]
CTRI	100.0 [68.1–100.0]	100.0 [68.1–100.0]	95.0 [73.2–99.7]	25.0 [12.8–42.5]
DRKS	- 1	- 1	- 1	- 1
NTR	- 1	- 1	- 1	- 1
CRiS	- 1	- 1	- 1	- 1
PACTR	- 1	- 1	- 1	- 1
RPCEC	- 1	- 1	- 1	- 1

Legend Table 6:

^1^ Less than 10 records, arms or outcomes, respectively.

To learn more about how data recording formats might influence data quality, data recording formats for contact details, interventions and primary outcomes were denoted for each of the registries that provided data to the WHO ICTRP at the time of the study (Table 7).

**Table 7:** Data recording formats for the three primary outcomes of this study (contact information, intervention specifics and outcome quality) at the registries that provided data to the ICTRP at the time of the study in 2013.

Data recording formats	Number of registries for which each question is 1			
	*true*		*false*	
*Contacts*				
Are there separate fields for scientific and public inqueries?	12	70.6%	5	29.4%
Are there separate fields for different contact details? And if so, a field for	17	100.0%	0	0.0%
the name of the contact person?	17	100.0%	0	0.0%
a telephone number?	15	88.2%	2	11.8%
an email address?	16	94.1%	1	5.9%
Is there a separate field for the Principal Investigator?	8	47.1%	9	52.9%
Is there a separate field for the person updating data?	3	17.6%	14	82.4%
Is there a separate field for the person that registered?	2	11.8%	15	88.2%
*Interventions*				
Are interventions categorized (e.g. drug, surgery, behavioural, etc)?	9	52.9%	8	47.1%
Are there separate fields for separate arms?	12	70.6%	5	29.4%
Does the intervention field contain specific sub-fields for different aspects of interventions? And if so, a sub-field for	10	58.8%	7	41.2%
arm label and/or description?	10	100.0%	0	0.0%
arm sample size?	3	30.0%	7	70.0%
arm type (intervention, active comparator, placebo)?	9	90.0%	1	10.0%
dose?	4	40.0%	6	60.0%
duration of the intervention?	2	20.0%	8	80.0%
frequency of administration?	1	10.0%	9	90.0%
route of administration?	3	30.0%	7	70.0%
*Outcomes*				
Are there separte fields for primary and secondary outcomes?	16	94.1%	1	5.9%
Are outcomes categorized (e.g. safety, efficacy)?	2	11.8%	15	88.2%
Does the outcome field contain specific sub-fields for different aspects of outcomes? And if so, a sub-field for	11	64.7%	6	35.3%
name?	11	100.0%	0	0.0%
time point?	11	100.0%	0	0.0%
method of measurement/measure specification?	2	18.2%	9	81.8%

Legend Table 7:

^1^ There is information on 17 registries in this table, instead of 15, because the JPRN registry is in fact a registry network that consists of three registries that all provide data to the WHO ICTRP. Each of these was assessed separately for this table.

## Discussion

### A persistent problem

This study was conducted using the same methods and the same research team as our previous study on the quality of registration.[2] There have been small but significant improvements in the quality of registration since 2009. However, important problems with quality remain and continue to constitute an impediment to the meaningful utilization of registered trial information.

There have been small improvements to the presence of contact details overall. This is partially due to the larger proportion of non-industry trials in the analysis of trials registered in 2012, which do better on registering contact details. But across all sponsor categories quality also improved, the main exception being the continued lack of mentioning of names of contact persons by industry sponsors. Explicit mentioning of the name of the principal investigator is important to increase the accountability of trialists. Furthermore, despite improvements, contact information such as a telephone number or email address often remain absent. Remarkably, trialists appear to remove contact details when trials have been completed or stopped, in particular for industry sponsored trials. To allow patients, healthcare workers and other researchers to inform themselves of clinical trials, it is important that trialists can be contacted at any stage of a trial. Such information should remain available after a trial is completed or stopped.

There was some improvement in the completeness of intervention specifics for drug trials, however, the improvement was minor. Contrariwise, the improvement in the quality of registered outcomes was marked. This is a hopeful development for systematic reviewers, since in the absence of a complete trial protocol, registered clinical trial data constitute the only way to identify selective reporting.[2], [13], [15]–[19] However, specific information about the outcome in registered records is necessary to detect selective outcome reporting as part of systematic reviews, and still almost half of all primary outcomes do not constitute a specific measure with a meaningful timeframe. Moreover, it has been proposed that the specificity of outcomes should be assessed at a greater level of granularity, to take into account more subtle forms of selective reporting.[30]

Finally, a very large percentage of records remains registered retrospectively, as has also been concluded by others.[50] Without prospective registration, before enrolment of the first participant, we cannot be certain that trial outcomes are not retrospectively registered in such a way that favours a particular result.

In conclusion, there have been small improvements to the quality of registered trial data, but poor quality is a persistent problem. Recent publications have also shown concomitant results reporting at individual registries to be problematically incomplete, such as at ClinicalTrials.gov [51]–[53], despite legal obligations in the US to report the findings of trials.[51], [54]

### The causes of poor quality (and learning from other registries)

The persistent nature of poor quality of registered clinical trial data suggests one or more pervasive causes. Although trialists themselves have a responsibility to ensure that the information in registered records is complete and accurate, registries can encourage high-quality registration through quality control processes and appropriate data recording practises.[2] Both are addressed in the International Standards for Clinical Trial Registries.[42]

Our analysis suggests that there are important differences between registries with regards to registration quality. Notably, there are few that score badly on all three aspects of quality that we tested, or well on all. Rather, there are differences depending on which aspect is assessed, as becomes clear from Table 6, and from our sensitivity analyses, which showed that the three largest registries score better on intervention and primary outcome quality, but worse on the presence of most contact details. One explanation for these differences is the variation in data recording formats between registries.[31] For example, some registries specifically ask trialists for the methods of measurement for each outcome. Others have only free text fields for outcomes. Some registries ask for specific details on interventions, others, again, have only free text fields. Some registries ask trialists to categorize interventions and outcomes, others do not. For data quality and data aggregation purposes,[23] it is important that discrete options are offered where there is a limited set of possible answers (supplemented by a free text field to allow for additional explanation where needed), that different sub-aspects of data set items are specifically queried (Table 7), and that the data recording formats are harmonized across all individual registries. A second explanation for the differences in the quality of registration between registries is the level of quality control that registries apply.

The differences in the quality of registration of different data items found in this study suggest that registries can learn from each other. Differences between registries in terms of data recording formats and their consequences for data quality deserve to be studied in more detail, so that registries can improve their formats based on the lessons from other registries. Registries could also draw lessons from each other about quality control, for example with regards to the information that is considered mandatory and a precondition for registration, and the different tiers of data checking (e.g. automated checks and manual checks [30], [42]) that can be implemented to detect incomplete or non-meaningful entries. The International Standards for Clinical Trial Registries state that benchmarking of registries should be one of the next steps in standards development for registries.[42]

### Enforcement

To be able to make use of the potential benefits that clinical trials registration offers, it is of paramount importance that registration is complete and accurate. However, it must also be comprehensive.[2] Enforcement of clinical trials registration has increased substantially over the past decade,[55] owing to national legislation on registration [4], [30], [56], policies by journal editors and publishers making registration a prerequisite for publication [1], [5]–[7], [57], ethics committees and national research ethics oversight agencies requiring registration as part of procedures for ethics approval [3], [55], [58], policies by funders making registration a prerequisite for grant approval [59], international codes of research practice that recommend trial registration, such as the SPIRIT 2013 and CONSORT 2010 statements which include sections recommending the admission of trial registration details to both clinical trial protocols and reports [60], [61], international codes of research ethics, such as the declaration of Helsinki [62], and self-regulation by universities [55] and the pharmaceutical industry [8]. Despite these measures, a proportion of trials currently remains unregistered, especially in countries lacking legislation on trial registration.[63]–[67]

National legislation is crucial in enforcing the registration of all clinical trials.[4] Several of the other enforcement measures outlined above have been instrumental in creating momentum for clinical trials registration, such as journal and ethics review board requirements for registration, yet not all journal editors require registration as a pre-condition for publication,[67] not all clinical trials are conducted with the goal of publication,[4] and not all ethics committees have policies on clinical trials registration in place [58]. Therefore, it is imperative that all countries that have not implemented legislation on trial registration do so.[4] Furthermore, it is important that the remit of legislation on registration should cover all possible clinical trials, as is being recognized in the US and the EU.[68], [69] Currently, in those countries where legislation to enforce registration is present, its remit is often limited to a sub-set of trials.[30], [68]

With regards to enforcement, the commitment of the pharmaceutical industry to clinical trials registration is important and the past development of a Joint Position of several pharmaceutical associations on the disclosure of clinical trial information via clinical trial registries and databases is laudable.[8] However, the Joint Position needs revisiting on three important aspects. First, currently, it allows for registration after commencement of patient enrolment. This is in contradiction with policies on clinical trial registration by WHO and the International Committee of Medical Journal Editors (ICMJE).[1], [2] Second, it allows trialists to withhold data specified by the WHO Minimum Trial Registration Data Set if they consider it sensitive. This, too, is in contradiction with policies on clinical trial registration by WHO and the ICMJE.[1], [2] Third, the Joint Position mentions that “registration of clinical trials on any one of a number of free, publicly accessible, internet-based registries should achieve the intended objectives”. To ensure the quality of registered trial data, the WHO ICTRP search portal only provides access to data from trials registered at registries that meet certain quality standards (excluding, for example, registries managed by for-profit agencies).[70] To realize a single point of access to all clinical trial data conducted globally, it is important that the pharmaceutical associations include a commitment to registration in WHO approved registries in the next update of their Joint Position, as the ICMJE already has.[7] Finally, enforcement of trial registration by the pharmaceutical industry would be further advanced if support for clinical trials registration and results reporting would not be limited to statements from the pharmaceutical associations, but if more individual pharmaceutical companies would subscribe to the AllTrials campaign, following the example of GlaxoSmithKline.[71]

Besides increasing the number of trials that is registered, enforcing measures could also help improve the quality of registration. Journal editors, for example, have been called upon to not only enforce registration itself, but to also implement quality control procedures.[19] Although editors have made clear that trial registration with missing or uninformative fields for the minimum data elements is inadequate,[1], [5], [57], [72] little is known about to what degree journals are putting such measures into practice.[6] Similarly, both in the EU and in the US legislature supports the WHO Minimum Trial Registration Data Set – the minimum amount of trial information that must appear in a register in order for a given trial to be considered fully registered.[73], [74] Failure to comply with registration legislation may result in penalties or withholding of federal grants.[54] Yet, little is known to what extent legislators are planning to invoke such measures, and whether the quality of registration could play a role in such decisions. For both legislators and journal editors discussion needs to be initiated on how far measures should go to discourage incomplete or inadequate registration. This applies to both the initial registration of a clinical trial, which was the subject of this study, as for results reporting in registry databases.[51]–[53]

### Conclusion

There have been small but significant improvements in the quality of registration since 2009. However, important problems with quality remain and continue to constitute an impediment to the meaningful utilization of registered trial information. More effort needs to be made to improve data recording formats, enhance quality control measures and scale up enforcement of trial registration.
